# Put on the sidelines of palliative care: a qualitative study of important barriers to GPs’ participation in palliative care and guideline implementation in Norway

**DOI:** 10.1080/02813432.2024.2306241

**Published:** 2024-01-30

**Authors:** Anne Fasting, Irene Hetlevik, Bente Prytz Mjølstad

**Affiliations:** a General Practice Research Unit, Department of Public Health and Nursing, NTNU, Norwegian University of Science and Technology, Trondheim, Norway; b Unit for Palliative Care and Chemotherapy Treatment, Oncology Department, Møre og Romsdal Hospital Trust, Kristiansund Hospital, Norway; c Saksvik legekontor, Saxe Viks veg 4, N-7562 Hundhammeren, Norway

**Keywords:** palliative care, primary care, palliative medicine, general practice, advance care planning, end-of-life care, transitions of care, clinical practice guidelines, Norway

## Abstract

**Background:**

Demographic changes, the evolvement of modern medicine and new treatments for severe diseases, increase the need for palliative care services. Palliative care includes all patients with life-limiting conditions, irrespective of diagnosis. In Norway, palliative care rests on a decentralised model where patient care can be delivered close to the patient’s home, and the Norwegian guideline for palliative care describes a model of care resting on extensive collaboration. Previous research suggests that this guideline is not well implemented among general practitioners (GPs). In this study, we aim to investigate barriers to GPs’ participation in palliative care and implementation of the guideline.

**Methods:**

We interviewed 25 GPs in four focus groups guided by a semi-structured interview guide. The interviews were recorded and transcribed verbatim. Data were analysed qualitatively with reflexive thematic analysis.

**Results:**

We identified four main themes as barriers to GPs’ participation in palliative care and to implementation of the guideline: (1) different established local cultures and practices of palliative care, (2) discontinuity of the GP–patient relationship, (3) unclear clinical handover and information gaps and (4) a mismatch between the guideline and everyday general practice.

**Conclusion:**

Significant structural and individual barriers to GPs’ participation in palliative care exist, which hamper the implementation of the guideline. GPs should be involved as stakeholders when guidelines involving them are created. Introduction of new professionals in primary care needs to be actively managed to avoid inappropriate collaborative practices. Continuity of the GP–patient relationship must be maintained throughout severe illness and at end-of-life.

## KEY POINTS OF THIS ARTICLE

According to the Norwegian guideline for palliative care, the GP should have a central position in providing primary palliative care.

Recent research and public reports suggest that not all GPs have such a central role or adhere to the guidelines.

This study highlights individual and structural barriers that could be addressed to increase GPs’ participation in palliative care and aid the implementation of the guidelines for palliative care.

## Introduction

The increasing incidence of cancer and the growing number of people living with life-limiting incurable diseases due to demographic changes increases the need for palliative care services [1]. Palliative patients need access to high-quality care [2,3]. Their conditions can change rapidly, demanding extensive cooperation between palliative care providers. An important outcome in palliative care is that patients are cared for and allowed to die in their preferred location, which is often their own homes [4,5].

Palliative care in Norway rests on a model in which patient care is provided in or near where the patient lives [2]. The rate of home deaths is less than 15%, and about 6.3% are likely to involve municipal end-of-life care at home, which is low in a European context [6–8]. Primary care provides basic palliative care with support from specialised palliative services at secondary and tertiary levels [2]. In Norway, the general practitioner (GP) is the primary point of contact for healthcare, and all citizens have the right to register with a regular GP serving as both coordinator of care and gatekeeper to secondary care [9]. The medical speciality of general practice is achieved by working as a GP under a 5-year curriculum and tutorial groups. The continuing medical education program (CME) includes group learning activities [10].

**Figure 1.**
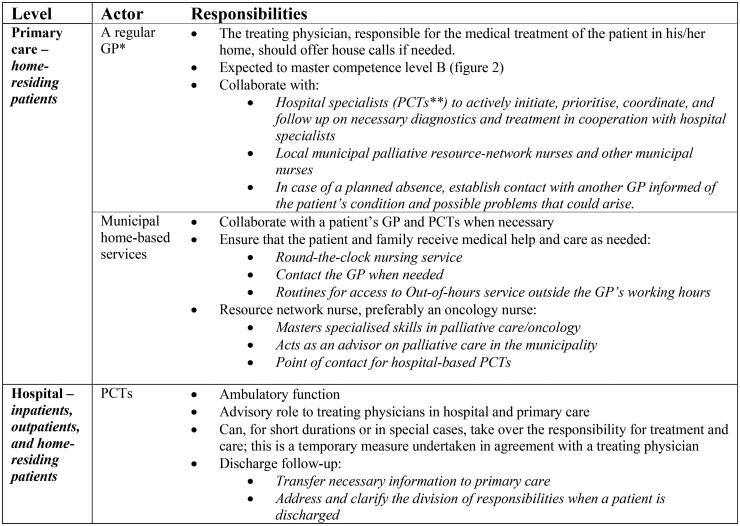
Extracts from a guideline showing the division of responsibilities between primary care and hospital-based services. *general practitioner **palliative care teams.

In Norway, the Directorate of Health is responsible for making national clinical practice guidelines, which today amount to more than seventy guidelines [11]. The guideline for palliative care in Norway, revised in 2015, was developed within the Norwegian national cancer care programs [2,12,13]. According to the guidelines, palliative care includes all patients with life-limiting illnesses [2]. The guideline contains clinical advice and a model for the organisation of palliative care, addressing competence levels for all health personnel and the allocation of responsibilities [2,14]. The guideline recommends hospital-based palliative care teams (PCTs) and municipal palliative care units. Municipal ‘network nurses’, preferably oncology nurses (ONs), should be organised in extensive resource networks [2]. Details of the organisation relevant to primary care are summarised in Figure 1, while a summary of the guidelines is in Additional file 1.

**Figure 2.**
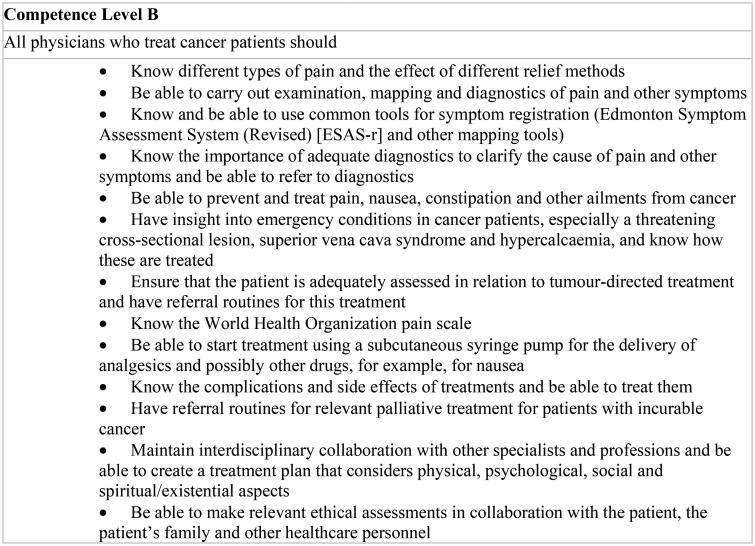
Extracts from the guideline showing level B competence.

The GP is central as coordinator of care, and the guideline contains specific competence requirements for GPs (see Figure 2). The PCTs should have an advisory role towards the GP and only take over responsibility for the patient in special cases. Consensus and public evaluations form the basis for these recommendations [2,12,15].

Previous research on guideline implementation shows that GPs find it difficult to adhere to guidelines, especially when different guidelines apply to single patients [16–20]. However, as palliative care is relevant in all life-limiting diseases, this guideline could serve as a trajectory for multiple conditions at end-of-life.

A national evaluation of Norwegian palliative care services in 2016 expressed concerns related to the lack of GP participation in palliative care [3]. Furthermore, previous research indicates that patients benefit from their GP participating in this work [21–25].

In a survey among regular GPs in one Norwegian county, we found that each GP has few palliative patients and limited experience with terminal care. The guideline was not well implemented, but half of the GPs perceived themselves as central in palliative care. Recommended working methods, such as patient-reported outcome measures (PROMs), were rarely used, possibly due to unawareness or reluctance to use such procedures [26].

We subsequently performed a focus group study to obtain knowledge about GPs’ experiences and role in palliative care. A previously published paper from this study showed that the GPs generally had positive attitudes toward palliative care and highlighted their strengths in this work. However, GPs’ involvement varied, and some struggled to find their role [27].

The aim of this study was to explore GPs’ experiences in order to gain new insights into barriers to their involvement in palliative care and implementation of the guideline.

## Methods

### Design

Exploring experiences and opinions warranted a qualitative approach [28]. The researcher adopts the role of both observer and participant in the dialogue with informants, requiring that preconceptions are made explicit [28]. Focus group interviews stimulate effective discussions among participants and provide easy access to several participants [28,29]. A semi-structured interview guide with open-ended questions was developed (see Additional file 2). GPs’ experiences with palliative care, including reflections about their role, were explored. They were thereafter presented with extracts from the guideline, and the descriptions of their role and competence requirements were discussed [27].

### Participants, setting, and data collection

Four focus group interviews were conducted in 2018. Each group chose a location where the group was interviewed once. The GP participants were from Mid-Norway, of both genders, varying ages, with different levels of professional experience, and from either group or solo practices. The GPs were purposively recruited from both urban and rural environments to represent typical Norwegian GPs. Four established CME groups of regular GPs and one tutorial group of GP trainees were contacted by author AF by mail or telephone. One CME group declined to participate. Written consent from all participants was obtained. Groups 1–3 were selected to represent three different municipalities affiliated with different PCTs. The tutorial group consisted of GPs from nine different municipalities and were affiliated with either the same PCTs as group 2 or 3 or a fourth local PCT.

A total of 25 participants, 15 males and 10 females were interviewed. The median age of the participants was 42. The mean patient list length was 1,032, and the mean amount of experience in general practice was 10.5 years. Table 1 summarises the demographic data of the groups.

**Table 1.** Characteristics of the participating GPs in groups (1–4). M = male and F = female.

**Group/** **Setting**	Gender	**Age** **range/** **mean**	Mean list length	Specialist GPs	**Experience as GPs range/** **mean**	Other previous or current occupational experience in the group
**G1** URBAN	3 M 1 F	41–48/ 44 years	1300	4	9–15/ 12 years	Nursing home physician, academic/PhD, and school physician
**G2** URBAN	3 M 2 F	45–67/ 53 years	850	3	7–39/ 20 years	School physician, nursing home physician, seafarers’ doctor
**G3** RURAL	4 M 3 F	33–61/ 44 years	1000	5	6–30/ 11 years	Children’s health care physician, nursing home physician, local medical officer, supervisor of intern doctors
**G4** RURAL AND URBAN	5 M 4 F	29–48/ 34 years	1000	1	1–15/ 4 years	Children’s health care physician, nursing home physician, officer of public health and communicable diseases, seafarers’ doctor

AF moderated all interviews accompanied by a researcher experienced in qualitative methods, who noted details of relevant non-verbal communication. AF wrote reflexive notes after each interview. The interviews were recorded on audio tapes and transcribed verbatim by AF, aided by the field notes. After the fourth interview, no new relevant themes emerged, the data was assessed as varied and well-suited for the purposes of the study, and the data collection therefore ended [30,31].

### Research group

A.F. works as a palliative care consultant in a hospital and is a specialist in general practice. B.P.M. and I.H. are experienced GPs and senior researchers with extensive expertise in the methods. Knowledge of the setting eased the authors’ access to the field of study. Our diversity of perspectives led to valuable insights in the research process. AF had no prior knowledge of the groups but was, by coincidence, professionally acquainted with some of the recruited participants.

### Analysis

We used reflexive thematic analysis by Braun and Clarke. The main aim of the thematic analysis is to identify meaning patterns from data of lived experience, guided by different phases. This includes repeated reading and familiarising with the data, searching for meaning and preliminary themes, and organising the findings in overarching patterns/themes [30,32]. Our approach was inductive and oriented towards both semantic and latent initial codes. Initial coding was handled with the NVivo software. Preliminary themes were formulated based on the generated codes. Subsequently, overarching themes were developed through discussion and reflections between A.F. and B.P.M. The transcripts were revisited to ensure the final themes corresponded with the original data. I.H. analysed the transcripts independently, and all three authors worked through the drafts and approved the final results. An audit trail was kept, and preliminary results were presented and discussed with peers from both academic and clinical environments.

## Results

The following themes were identified as main barriers to GPs’ participation in palliative care and to implementation of the guideline: 1) different established local cultures and practices for palliative care, 2) discontinuity of the GP–patient relationship, 3) unclear clinical handover and information gaps, and 4) mismatch between the guideline and everyday general practice.

### Different established local cultures and practices for palliative care

The GPs were generally unaware of the guidelines and unfamiliar with the content of the excerpts presented to them during the interviews. Some GPs, especially from rural areas, confirmed that palliative care in their municipality was organised in accordance with the guideline and that GPs had a central role as coordinators. These GPs saw the ON, if available, as an integral partner; furthermore, there was a close collaboration between the PCTs, ON and GP. Those GPs who had no ON said they collaborated closely with PCTs and municipal nurses. These GPs agreed with the GP role and tasks described in the guideline. This participant described how the GP provides basic safety for everyone involved:

- Male GP, group 3*: I believe the regular GP’s role is the key to the entire system. The municipal home care nurses are hesitant if they don’t have a regular GP they can contact when the need arises, and the role of the GP is to maintain close contact with the palliative care team in case they are needed for matters involving medication or the like, or as a point of contact for the palliative care team, who can facilitate contact between them and the municipal nurses.*

Other participants described an organisation of palliative care not in line with the guideline. Some did not know if an ON was available in their municipality, and several GPs, especially in urban practices, experienced that PCTs ‘took over’ general responsibility for patients. That was an important barrier to participation in palliative care, leading several GPs to question the relevance of the guideline. Some even wanted to discard the guideline due to the discrepancy between recommendations and perceived reality, as evidenced by this quote:

- Male GP, group 2: *The last point is especially important in this context [GP refers to the guideline]: ‘the GP is responsible for coordinating the patient’s medical treatment.’ If that were the point of departure, it would have been quite difficult. However, it says here that in particular cases, the hospital’s palliative team can take over the responsibility for treatment and, of course, that is what actually happens, so it [the guideline] isn’t particularly relevant for us.*

### Discontinuation of the GP–patient relationship

Several GPs reported discontinuation of the doctor–patient relationship when patients were referred to the hospital for severe disease, particularly for cancer. Patients with whom the GPs previously had regular contact were now cared for by others, even tasks that were usually within the GPs’ responsibilities. This was an essential reason for GPs not to be involved in palliative care. In the following illustrative case, a GP refers to a patient with schizophrenia and diabetes where the relationship was discontinued after the patient was referred:

- Female GP, group 1: *The patient had come to see me fairly regularly every month, and I felt we had a good connection. Then things changed abruptly when he got cancer. Now, he is immersed in the palliative system, and I feel like I have, in point of fact, lost contact with him. He spends all of his energy, and his time, in meetings with the Specialist Health Service. Then patients come to us when they experience something or other that is acute, don’t they? But the good conversation … I do my best, but I don’t actually know where he is in the system at the moment…*

Actively initiating contact with the patient was discussed in all the groups as a method of overcoming the discontinuation of the GP–patient relationship. While some GPs, typically from rural areas, advocated such approaches supporting the guideline’s requirement to ‘actively take a lead’, others pointed to structural barriers to doing so. The lack of tradition for outreach activities in general practice was particularly highlighted, as pointed out in this passage:

- Male GP group 2*: I find it much easier to establish contact if you are asked to make a house call, than if you were to initiate contact yourself, yes.*
- Female GP group 2*: The thing is, and there is something to this, GPs do not engage in proactive initiation of contact!*

### Unclear clinical handover and information gaps

Another barrier to GPs’ involvement was uncertainty about who was in charge of medical care when the patient was discharged to the municipality. Some PCTs continued their follow-up of patients after discharge. When PCTs had taken overall responsibility for home-residing patients, the GPs were uncertain about what role they themselves had:

- Male GP group 4: *I find that difficult because the palliative care team sometimes takes over the whole show, so to speak. They take over everything. Where does that leave us? What is our role? Have we no part to play then? Is our role just to get a phone call after hours when the morphine pump stops working and answer the question ‘what should we do now?’*

Several GPs found it difficult to fulfil the coordinator role. They could lose track of the patient’s disease trajectory, which, in turn, made it difficult to coordinate care, as explained by this GP:

- Female GP, group 2: *When a patient has a serious disease, and in particular when palliative care is involved in the final phase of life, you lose your patient. In other words, you lose the contact you had as the patient’s GP. You don’t know how far they’ve progressed in their treatment. Of course, you get their case history summaries, but it’s really hard to keep up with their progress on an ongoing basis…*

The GPs also stressed that they needed *timely and sufficient information* from the hospital to provide care. The following statement shows how an unclear handover could make it difficult for a GP to act:

- Male GP, group 1: *And then she [the patient] tells you that the hospital staff says that you are now supposed to be responsible for her follow-up. ‘Okay, what am I supposed to do then?’ I asked. Nope, she has no idea. And there is no mention of this in any of the medical reports. In other words, this is a rather vague order. What is more, she may have misunderstood the timing, so that I am only supposed to take over further out in her treatment. But there is no plan available before that. Following up on such a general basis [is difficult].*

A *realistic prognosis* provided by hospital specialists was vital for the GPs. Discharge papers were perceived by several GPs to contain redundant information on some aspects, while essential information for dealing with the complex aspects of care was lacking:

- Male GP, group 3: *We could have achieved much better trajectories for our patients if only we all had been given a realistic prognosis by the hospital. In other words, if the hospital had made the prognosis clear to the patient so that the patient was also prepared [for the inevitable outcome], then we would not have to deal with all the uncertainty, that is, that the patient believes there is a chance that he might recover. Often, we come along at that point, and we don’t know whether or not he is aware that he is going to die.*

Several GPs had experience with structured individual care plans and advance care planning (ACP). These were seen as effective tools for filling information and knowledge gaps, helping the GPs in their coordinator role. ACPs were particularly useful in out-of-hours decision-making. The GPs also appreciated physical meetings with patients and the healthcare personnel involved:

- Male GP, group 4: *I never thought I would say this, but I’m actually developing a liking for meetings. Quite simply because getting written information is different from getting a verbal briefing. Nor is it possible to communicate well enough in writing with all of those involved in the patient’s trajectory. Naturally, I’ve had some pastries and coffee with palliative patients. Even though little actually happens during such meetings, they are very good because they make my life and yours, and the life of the patient far easier. Simply being there can vastly improve our shared understanding of the situation.*

### Mismatch between the guideline and everyday general practice

GPs in all focus groups discussed the guideline in relation to their experiences with palliative care. Several concluded that the guideline had probably been designed without involving GP stakeholders. Aspects that were highlighted were pressure on GPs’ resources, the extensive expected competence level and the guidelines’ suitability for general practice. The GPs described palliative care as time-consuming, conflicting with their busy schedules:

- Female GP, group 2: *and it is clear that if you had had 4–5 patients at the palliative stage, you would have felt like you were drowning if you had followed the guideline to the letter. It would simply not be possible. It’s hard enough to have one patient who needs you that way.*

The requirements regarding GPs’ level of competence in palliative care were considered to go beyond what one might expect from a GP who needs to possess generalist competence in many fields. Learning specialised procedures was demanding, as explained by this GP:

- Male GP, group 4*: Obviously, we have expertise in some areas, but not in others. That’s true. For example, every time I see one of those morphine pumps, there is a new and improved model … And when they [the municipal nurses] tell you that it’s time to order morphine for it, well, I don’t know how to do that, so then I have to ring someone who does know how… When it comes to technical devices, I find that as GPs, it is virtually impossible to keep up with all the changes.*

The working methods described lacked flexibility compatible with the GPs’ approach. The GPs were reluctant to use guideline items such as the ESAS-r, which appeared rigid and was one of many different assessment forms they were expected to use. Guideline recommendations that did not seem to fit with the working methods and ‘language’ of general practice were questioned:

- Male GP, group 1: *There is a phrase that I don’t like. When they write, ‘Make a treatment plan’, perhaps I just react to the phrase because, of course, I have ideas about how I prefer to do this, but to sit down and write a treatment plan that takes into account ‘…physical, mental, social, spiritual, and existential aspects’, is beyond me. Meeting people at their particular stage of life, and even more importantly as they are approaching death, talking about death, talking about the fear of dying, these are the types of things I would rather do. In this context, there is something or other with formal language that is simply not entirely adequate.*

In all the interviews, GPs found the guideline chapters directed at symptom treatment useful. As previously mentioned, the GPs were generally unaware of the guideline. Only a few used it actively. This seemed connected to the GPs’ working methods: the extensive format and difficult accessibility of this guideline were problematic, making it ill-equipped to serve GPs’ needs. The GPs preferred more accessible handbooks or to search the internet to get answers quickly for specific clinical problems.

- Male GP, group 1: *Of course, we often have questions about specific cases, for example, we have a patient who is in pain, and we wonder how best to deal with pain management. Naturally, none of us has time to sit down and read a book to find answers. We have to figure things out quickly. This means that rapid access to knowledge and information is imperative. And in this type of situation…as the format for an educational tool, the guideline is useless in my opinion.*

## Discussion

### Main findings

The study identified both structural and individual barriers to GPs’ involvement in palliative care and guideline implementation. Whereas the guideline describes a decentralised model of care with the GP as coordinator, different local cultures of collaboration between primary care and specialised palliative care exist. PCTs who took over full responsibility for patients appeared to contribute to putting the GP on the sidelines of palliative care. This was a barrier to GP participation and an important reason for GPs to question the guideline’s relevance in general practice. Discontinuity of the GP–patient relationship was frequent and could prevent GPs from adopting a central role for palliative patients. Moreover, experiences of unclear clinical handover and information gaps made it difficult for GPs to coordinate care or even recognise the patient as palliative or at end-of-life. These structural barriers were most prominent in the accounts of GPs from urban environments. The GPs questioned whether there had been sufficient GP participation in creating the guideline and pointed to a mismatch between the guideline requirements and the realities of general practice. At the individual level, unawareness of the guideline, behaviours such as using other sources for decision support, and negative attitudes to guidelines in general were most prominent.

The guideline chapters aimed at symptom treatment and recommendations for joint meetings and individual care plans were appreciated by the GPs.

### Reflexivity, strengths and limitations

Conducting and analysing focus group interviews warrants close attention to the researcher’s preconceptions that may influence which topics are pursued during the interviews and how the material is interpreted. Several steps were taken to increase trustworthiness. Using a well-described method of analysis and keeping an audit trail of decisions and extensive reflexive activity added to the dependability of results. Presenting and discussing preliminary findings with peers and fellow researchers added to the credibility of our results. To aid the transferability of the results, we provide rich details on the study setting and participants and use direct quotes in the results section. Throughout the analysis and writing of the manuscript, we reflected on the ten common problems in TA research raised by Braun and Clarke [30].

Recruiting existing CME/tutorial groups contributed to minimising problems associated with self-selection of GPs with a special interest in the topic.

Limiting recruitment to one geographic region introduced issues of representability. At the same time, it facilitated a strategic, representative sampling within a typical Norwegian county. We believe that our informants’ views may be representative of many Norwegian GPs and thus transferable to similar settings.

### Findings in light of current knowledge

#### The evolvement of different local cultures for palliative care:

The results of this study indicate that established local cultures of collaboration in palliative care seemed to vary between municipalities and were closely linked to the professional relationships between the GP, PCTs and ONs. According to the guideline, the GP is responsible for medical treatment and coordination of care, but the ON is also assigned a coordinating role and serves as the point of contact for PCTs (Figure 1) [2]. Exactly how this *PCT, ON and GP triad* should collaborate is not specified and seems to be left to the discretion of either the ON or the PCT [2]. Our findings could indicate that direct contact between the ON and the PCT creates a bypass of the GP, which is in line with other studies [26,33,34]. It could be interpreted as the PCTs and ONs not following the guideline recommendations, pushing the GPs to the side. In Norway, the establishment of municipal ONs as coordinators of health services for cancer patients is growing [35]. This ON role is based on the specialised ‘pivot’ nursing role in oncology developed in Canada [36], allowing for professional autonomy similar to that of the nurse practitioners [37]. The introduction of nurse practitioners is an essential part of healthcare reforms in many parts of the world, whereas optimal integration of them into the existing structure may be challenging, in line with our findings [37,38]. A preference for the specialised resource network nurses to be ONs is expressed in the guideline [2]. Consequently, palliative patients with non-cancer diagnoses, whose palliative care needs could be equally challenging for the GPs, do not automatically benefit from the recommended care arrangements.

Individual PCTs’ propensity to ‘take over’ and provide total care for their patients could not fully explain differences in culture, as GPs from different municipalities affiliated with the same PCT could give contradictory descriptions of how the PCT team acted. The different cultures seemed to follow a pattern consistent with previous findings showing that rural GPs are more involved in palliative care than their urban colleagues [26,27, 39]. Although this could mean that rural residents benefit from the close involvement of GPs, the finding must also be seen in light of the fact that the demands for specialised palliative care services in Norway exceed the existing resources [3]. A recent systematic review of studies from different countries has shown a connection between rural residency and the under-use of specialised palliative services for elderly cancer patients [40]. Therefore, our findings may also be interpreted to mean that rural areas have less access to specialised palliative care than urban areas, as found in other studies [41, 42].

#### Discontinuity of the GP–patient relationship, clinical handover, and information gaps:

Our results indicate that it is common for palliative patients to lose contact with their regular GP. Similar findings have previously been obtained for cancer patients in various settings and countries [42–45]. Continuity of care is perceived as one of the greatest strengths GPs can contribute to palliative care [27]. In a systematic review from 2016, cancer patients were found to appreciate the continuous involvement of GPs to provide primary healthcare and help the patients ‘negotiate the system’ [42]. A Danish study published in 2011 identified the GP as the ideal ‘key worker’ in palliative home care [22].

There was no consensus among the GPs on whether they should take an active role in relation to the patient. This is in line with a Dutch study on the GP’s role in cancer survivorship, showing that GPs tend to adopt a reactive approach, expecting patients to make contact and clearly present their complaints [46]. However, according to a recent systematic review, proactive strategies by GPs improve patient outcomes [47]. We also found that clinical handover was a barrier as it was unclear to many GPs whether they were expected to be involved. A recent systematic review of clinical handover and handoff points to the multiple hazards to patient safety that may follow an unclear handover [48]. In palliative care, the transition from hospital to home-based care is particularly vulnerable [49]. The discharge letters are essential for cooperation, and ambiguities in allocating responsibilities and inefficient communication are not uncommon [50–52].

Our informants highlighted the benefits of participating in joint meetings to optimise the flow of information. Such interdisciplinary meetings are an important part of discharge planning in palliative care, although they seem underused [26, 53,54]. Several of the GPs appreciated the use of individual care plans and ACPs, which are not consistently implemented in practice [26]. ACP is internationally recognised as a tool to improve medical care for chronic and life-threatening diseases [55]. ACP in primary care increases time at home and the number of deaths at home for cancer patients [56]. Furthermore, a recent systematic review demonstrated superior patient outcomes in palliative care when GPs were involved in decision-making [57].

#### Map versus terrain: exploring the mismatch between the guideline and everyday general practice

The overall impression was that several of the guideline’s demands and items were unsuitable for general practice, and the model of care suggested by the guideline did not fit the existing culture for palliative care everywhere. This mismatch was clearly a structural barrier to the implementation of the guideline. There is a complex negotiation within a real-life context in which a guideline is to be implemented, and pragmatic considerations can be decisive in implementation in general practice [58,59]. Clinical guidelines are designed to translate evidence into practice, optimise patient care and avoid variation in service provision [60]. According to Lau et al. implementation studies typically focus on interventions on the individual level and fail to address implementation issues in a broad context or look at the barriers relevant to the setting [61]. Primary care has more complex needs than what is considered in many guidelines, which may be linked to cultural differences between the specialists leading guideline creation and the primary care personnel who have other needs [62–64]. Our participants questioned whether GPs participated in creating this guideline. Although the creation process of this guideline meets several of the quality criteria for clinical guidelines [60,65,66], it rests on low levels of evidence [2]. Although consensus development is recognised in guideline development, specialists in a field typically focus on recommending the techniques known to them and are unaware of other approaches [65,67]. The GPs’ reluctance to use the PROMs suggested in the guideline must be seen in the light of this and agrees with suggestions that PROMs in general practice should be selected based on the needs of primary care clinicians in a bottom–up approach [68]. Including GPs as stakeholders in guidelines that apply to them has previously been shown to be important [65,69]. Despite several GP specialists having participated at various points in the creation of this guideline, the GPs in our study found that the guideline requirements did not fit with the realities of general practice.

Our findings thus add to previous arguments that GPs who do not adhere to guidelines may have valid reasons for not doing so [70].

## Conclusion

Our study provides essential information about structural barriers that need to be considered when creating and implementing guidelines involving general practice. Appropriate and sufficient stakeholder involvement from GPs is vital to ensure that guideline requirements harmonise with the realities of general practice.

Unclarities in this guideline could lead to different interpretations and views on the appropriate division of responsibilities, which may give rise to practices incompatible with the model of care intended. As a result, GPs can be pushed to the sidelines of palliative care. New professional roles need managing and integration into the existing structure of primary care to avoid inappropriate practices that weaken the GP’s gatekeeper function, as we have demonstrated in this study.

Clear communication addressing the division of responsibilities and increased use of individual care plans, ACPs, and collaborative meetings agree well with the GPs’ clinical reality and could optimise collaboration between GPs, ONs and PCTs. Steps should be taken at all levels of the health care service to maintain the GP–patient relationship throughout severe illness and at end-of-life.

## Supplementary Material

Supplemental Material

Supplemental Material
