# Supplementary material for: Put on the sidelines of palliative care: a qualitative study of important barriers to GPs’ participation in palliative care and guideline implementation in Norway
Source: Scand J Prim Health Care. 2024 Jan 30;42(2):254–65. doi: 10.1080/02813432.2024.2306241 (PMC11003325; doi:10.1080/02813432.2024.2306241)
Supplement: Supplemental Material [file IPRI_A_2306241_SM7039.docx]

Summary of the 2015 revision of the Norwegian guideline for palliative care

**Who is covered by the action program**

The action program takes upon treatment, nursing, and care for patients with uncurable cancer and limited life expectancy. In some recommendations the palliative patient population is limited to include patients with life expectancy of less than 9-12 months. In reality the palliative phase is a process starting when it is acknowledged that the disease is incurable, and that ends when the patient dies. Palliative care also includes mourning and follow-up of relatives.

Much of the available knowledge and documentation in palliative care is linked to cancer patients. De general conditions described in this action program, will however also be valid for palliative patients with other diagnoses. Palliative care is performed within most medical fields and at all levels of the health service.

**Chapter 1. Introduction**

This chapter describes the basic features of palliative care, such as the patient population, treatment culture and the basic elements of investigation and treatment. All patients with serious, advanced disease have a right to necessary medical aid, and therefore falls within the priority regulation.

**Chapter 2. Characteristics and challenges of palliative care**

The chapter describes the contents of palliative care work and conditions of the palliative population that requires special competence. The contents are based on established professional practices both nationally and internationally, and for the subjects where grading of evidence is found possible and relevant, this is undertaken.

**Chapter 3. Trajectory times**

In the course of 2015, 28 trajectories for cancer will be implemented, including trajectory deadlines for the different cancer diagnoses. These deadlines will replace the existing limits of 5, 10 and 20 working days.

**Chapter 4. Symptoms and conditions**

The chapter consists of clinical guidelines for investigation and treatment of central symptoms and conditions in palliative care. The guidelines mainly concern patients with cancer. The general level of evidence in palliative treatment is deficient and often based on experience.

**Chapter 5. Implementation of the recommendations**

The chapter presents propositions as to how the action program’s recommendation for organization, education, competence, and professional quality can be implemented.

**Chapter 6. Process and method for the preparation of the guideline.**

In this chapter the National centre of healthcare knowledge (Kunnskapsssenteret) has described the methods involved in the preparation of the action program.

**Appendix 1. Organization**

In this appendix a standard for the palliative care service in the various organizational units in hospital and primary care is presented. The tasks of each unit are described together with the requirements for the clinical service provision and organization, including involved personnel, premises, and equipment. The recommendations rest on public evaluations and reports, and the document “Standard for palliative care”.

**Appendix 1. Competence requirements**

Optimal palliative care warrants that all personnel have the necessary professional competence. This includes knowledge, skills, and attitudes.

In this appendix requirements for the competence of relevant groups of professionals (nurses, doctors, physiotherapists, social workers, clinical nutritionists, occupational therapists, auxiliary nurses, priests, psychologists, and other relevant professionals within palliative care) and how this competence currently can be achieved. The working group also promotes several proposals for measures to increase competence in palliative care.

The account is based on the following division of competence levels:

**Level A:** Basic competence. This should be mastered by all health workers within the relevant groups of professionals and must be taught in the in the basic education.

**Level B:** Necessary competence for health professionals who treat palliative patients as part of their clinical work, both in the community and in hospitals.

**Level C:** Necessary competence for health workers that work in palliative teams and palliative units. This level equals specialist competence.

**Appendix 3. Relevant web-sources**

Provides a list of relevant web-sources for various parts of palliative care.
